# Praja1 E3 ubiquitin ligase promotes skeletal myogenesis through degradation of EZH2 upon p38α activation

**DOI:** 10.1038/ncomms13956

**Published:** 2017-01-09

**Authors:** Silvia Consalvi, Arianna Brancaccio, Alessandra Dall'Agnese, Pier Lorenzo Puri, Daniela Palacios

**Affiliations:** 1 Laboratory of Epigenetics and Regenerative Pharmacology, IRCCS Fondazione Santa Lucia, Via del Fosso di Fiorano, 64, 00143 Rome, Italy; 2 Laboratory of Epigenetics and Signal Transduction, IRCCS Fondazione Santa Lucia, Via del Fosso di Fiorano, 64, 00143 Rome, Italy; 3 Department of Anatomy, Histology, Forensic Medicine and Orthopedics, Via Scarpa 14, Sapienza University, 00161 Rome, Italy; 4 Sanford-Burnham-Prebys Medical Discovery Institute, Development Aging and Regeneration Program, La Jolla 92037, California, USA

## Abstract

Polycomb proteins are critical chromatin modifiers that regulate stem cell differentiation via transcriptional repression. In skeletal muscle progenitors Enhancer of zeste homologue 2 (EZH2), the catalytic subunit of Polycomb Repressive Complex 2 (PRC2), contributes to maintain the chromatin of muscle genes in a repressive conformation, whereas its down-regulation allows the progression through the myogenic programme. Here, we show that p38α kinase promotes EZH2 degradation in differentiating muscle cells through phosphorylation of threonine 372. Biochemical and genetic evidence demonstrates that the MYOD-induced E3 ubiquitin ligase Praja1 (PJA1) is involved in regulating EZH2 levels upon p38α activation. EZH2 premature degradation in proliferating myoblasts is prevented by low levels of PJA1, its cytoplasmic localization and the lower activity towards unphosphorylated EZH2. Our results indicate that signal-dependent degradation of EZH2 is a prerequisite for satellite cells differentiation and identify PJA1 as a new player in the epigenetic control of muscle gene expression.

## Abstract (web summary)

In skeletal muscle progenitors, EZH2 maintains myogenic genes in a repressed state, but during differentiation its levels are reduced via unknown mechanisms. Here the authors show that during myogenesis, p38α kinase phosphorylates EZH2 and targets it for degradation by the ubiquitin ligase PRAJA1.

Muscle regeneration is a multi-step process stimulated in response to injury, that begins with the activation of a population of muscle stem cells called satellite cells^1^,^2^. After an initial expansion, activated muscle progenitors exit the cell cycle and terminally differentiate through a series of events that entail the coordinated activation and repression of discrete subsets of genes^3^. Amongst the epigenetic modifiers that regulate gene expression in stem and progenitor cells are Polycomb proteins. Of them, Enhancer of zeste homologue 2 (EZH2) methylates lysine 27 of histone H3 (H3K27me3), a hallmark of Polycomb-mediated gene repression^4^,^5^. Work from the past few years suggests that EZH2 plays a key role in muscle regeneration by repressing gene expression at different stages of the transition from activated muscle progenitors to differentiated cells^6^,^7^,^8^. Two different studies using conditional knock out mice have highlighted the importance of EZH2 in maintaining the self-renewal and proliferation of satellite cells, showing that genetic ablation of *Ezh2* in satellite cells leads to a decrease in stem cell number and impaired muscle regeneration^9^,^10^. Interestingly, EZH2 levels dramatically decrease upon differentiation of muscle cells, being barely detectable in fully differentiated myotubes^8^. Several molecules have being implicated in the transcriptional and post-transcriptional regulation of the gene in normal and tumour cells, including members of the E2F family of transcription factors^11^, p53 (ref. 12) and small non coding RNAs^7^,^13^,^14^. However, less is known on the signals and post-translational mechanism that modulate EZH2 protein levels during somatic cells differentiation.

Recently, the identification of EZH2 as a nuclear phosphoprotein that integrates information from intrinsic and extrinsic cues suggested that Polycomb Repressive Complex 2 (PRC2) activity, distribution and homeostasis can be regulated by a number of signalling cascades^15^. Of the signalling cascades affecting PRC2 function, we previously showed that p38α mitogen activated protein kinase (MAPK) directly phosphorylates human EZH2 on threonine 372 (T372). p38α-mediated phosphorylation relocates EZH2 to *Pax7* promoter to repress its expression in satellite cells induced to differentiate, an event that is necessary for cell-cycle exit^6^,^16^. p38α, which is activated by inflammatory cues in regenerating muscles, plays a fundamental role in regulating gene expression during muscle differentiation^6^,^17^,^18^,^19^,^20^,^21^,^22^,^23^. As p38α activation occurs at the onset of myogenic differentiation, when EZH2 levels start to decrease, we speculated p38α signalling could be involved in regulating EZH2 levels at early stages of muscle differentiation. Here we demonstrate that p38α regulates EZH2 protein stability, marking it for proteasome-mediated degradation. Furthermore, we identify the E3 ubiquitin ligase Praja1 (PJA1) as a novel component of the myogenic programme involved in EZH2 degradation upon activation of the p38α cascade.

## Results

### Phosphorylation of T372 marks EZH2 for degradation

**Figure 1:**
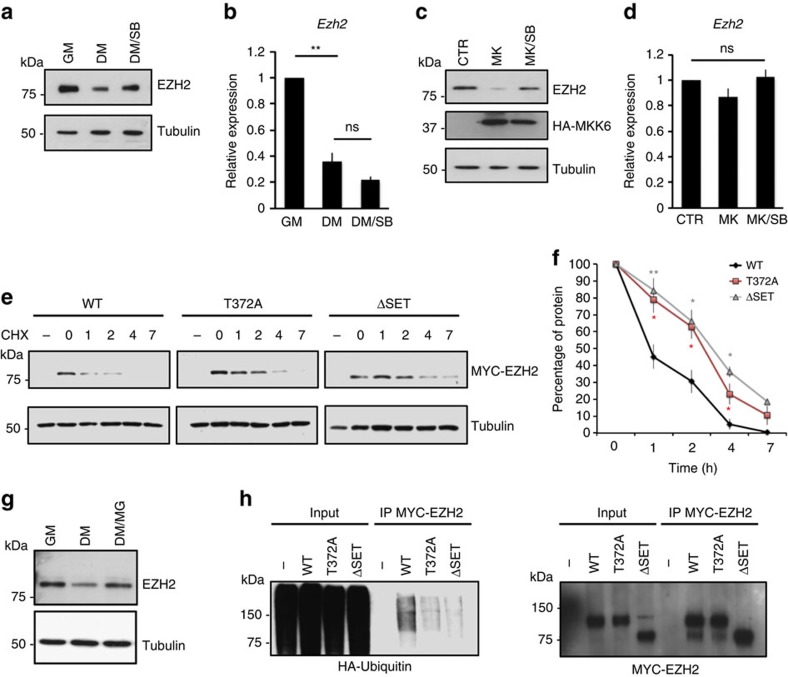
p38α kinase modulates EZH2 stability during skeletal myogenesis. (**a**) Western blot and (**b**) qRT-PCR analysis of EZH2 levels in C2C12 cells cultured in GM or induced to differentiate in the absence (DM) or presence (DM/SB) of the p38α/β inhibitor SB202190 (SB). Tubulin is shown as a loading control. (**c**) Western blot and (**d**) qRT-PCR analysis of EZH2 levels in C2C12 cells infected with control (CTR) or *MKK6EE-*containing adenoviruses and cultured in the absence (MK) or presence (MK/SB) of SB. (**e**) Stability assay performed on C2C12 cells transfected with either wt, phospho-mutant (T372A) or catalytically inactive (ΔSET) versions of myc-tagged *Ezh2* constructs and induced to differentiate for the indicated hours in the presence of cycloheximide (CHX). First lane in each panel correspond to untransfected cells. (**f**) Graph representing the relative amount of EZH2 measured by western blot in **e** and normalized against tubulin at the different time points. (**g**) Western blot showing EZH2 levels in C2C12 cells cultured in GM or induced to differentiate in the absence (DM) or presence (DM/MG) of the proteasome inhibitor MG132. (**h**) Ubiquitin levels associated to the indicated EZH2 constructs were measured by western blot using an antibody against HA after co-transfection of C2C12 cells with myc-*Ezh2* and HA-*Ubiquitin* and immunoprecipitation using an anti-MYC antibody. MG132 was added to the cells 2 h before collecting to avoid degradation of ubiquitinated MYC-EZH2. Input proteins (10% of immunoprecipitated material) are also shown. Data are represented as mean±s.e.m. of three independent biological replicates. *P<0.05; **P<0.01, ns, not significant (Student's test).

To investigate if and how p38α signalling regulates EZH2 levels during myogenesis we first performed western blot (Fig. 1a) and qRT-PCR (Fig. 1b) analysis on C2C12 muscle cells incubated in growth medium (GM) or induced to differentiate in differentiation medium in the absence (DM) or presence (DM/SB) of the p38α/β inhibitor SB202190. Of note, incubation in DM induces phosphorylation of EZH2 on T367 (corresponding to T372 in human) and this phosphorylation is impaired in the presence of SB202190 (Supplementary Fig. 1a). Our data show that, whereas *Ezh2* transcription is not altered by SB202190, there is a partial recovery of EZH2 protein levels upon SB202190 treatment. We performed similar experiments in cells incubated in GM and infected with a constitutively active form of the upstream mitogen activated kinase kinase 6 (MKK6), MKK6EE. Our results indicate that MKK6EE over-expression destabilizes EZH2 protein in proliferating myoblasts (Fig. 1c) without altering transcription of the gene (Fig. 1d) and this is reverted in the presence of SB202190. Given that MKK6EE and SB202190 modulate the activity of both p38α and p38β our results do not exclude the possibility p38β is involved in this response. However, recent data showed that p38β is barely expressed in differentiated C2C12 cells^24^, pointing out to p38α as the kinase modulating EZH2 protein stability during muscle differentiation. To investigate if phosphorylation of the specific residue T372 marks EZH2 for degradation we performed protein stability assays in the presence of cycloheximide on C2C12 cells over-expressing constructs containing either wild type (wt) or a phospho-mutant version of the protein (T372A). Given that little is known on the regulation of EZH2 stability and its correlation with the histone methyltransferase activity, we also included in the analysis a catalytically inactive mutant lacking the SET domain (ΔSET). The data show that substitution of T372 with a non-phosphorylable residue (T372A mutant) or deletion of the catalytic domain increases protein stability as compared with wt protein (Fig. 1e,f).

We next investigated the mechanism through which p38α controls EZH2 levels in muscle cells. The ubiquitin proteasome system (UPS) is a conserved pathway of controlled proteolysis that initiates with the ‘marking' of target proteins through the post-transcriptional addition of the 76 aminoacids protein ubiquitin^25^. Pharmacological inhibition of the proteasome through the use of MG132 indicates that EZH2 is targeted to proteasome-mediated degradation during muscle differentiation (Fig. 1g). By using the T372A and ΔSET EZH2 mutants we demonstrate that both phosphorylation of T372 and the integrity of the SET domain are necessary for EZH2 ubiquitination and degradation when cells are cultured in DM (Fig. 1h and Supplementary Fig. 1b). In addition, replacing the T372 with a negatively charged aminoacid that mimics phosphorylation (T372D) anticipates EZH2 degradation in proliferation conditions (Supplementary Fig. 1c). Of note, the increased stability of the EZH2ΔSET construct is not due to impaired phosphorylation of the mutant, as our published data show that a mutant lacking the SET domain is still phosphorylated by p38α (ref. 6). Nor to impaired methyltransferase activity per se, as treatment of cells with an EZH2 inhibitor, GSK-126, does not modulate protein stability (Supplementary Fig. 1d).

Altogether, the data presented here suggest that p38α kinase modulates EZH2 stability via phosphorylation of T372 and UPS targeting.

### PJA1 is a novel component of the myogenic programme

**Figure 2:**
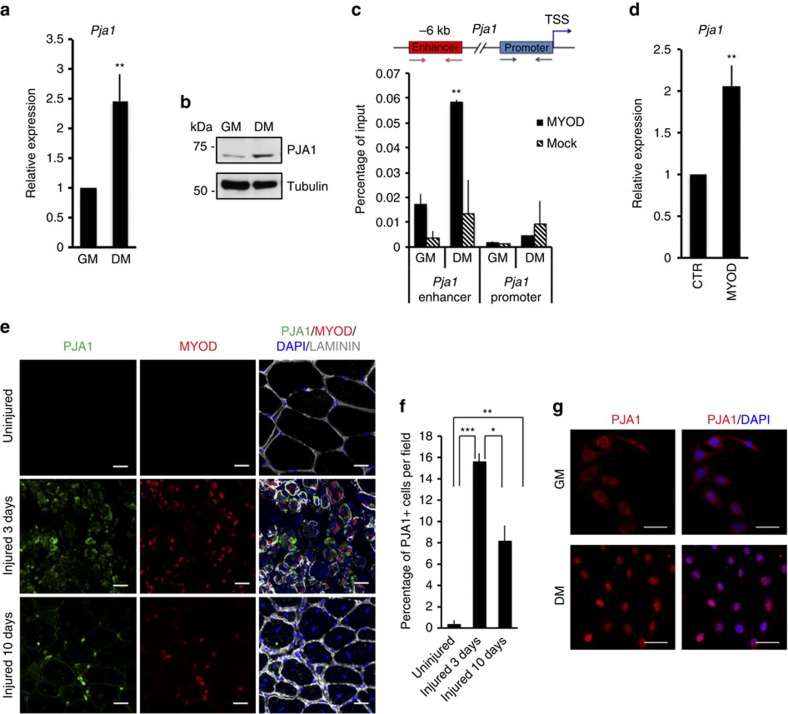
The E3 ubiquitin ligase *Pja1* is induced by MYOD during muscle differentiation. (**a**) qRT-PCR and (**b**) western blot analysis showing PJA1 levels in C2C12 cells cultured in GM or induced to differentiate for 18 h in low serum-containing medium (DM). (**c**) ChIP assay performed in C2C12 cells treated as in **a** using an antibody against MYOD. Mock IP is included as a negative control. Graph shows qPCR amplification at a regulatory region upstream *Pja1* TSS and at the TSS (negative control) on immunoprecipitated material normalized against input DNA. (**d**) qRT-PCR data showing *Pja1* expression on BJ fibroblasts infected with control (CTR) and *MyoD*-containing adenovirus and cultured in DM for 48 h. (**e**) Immunofluorescence using antibodies against MYOD, PJA1 and laminin on transversal sections of tibialis anterior (TA) muscles derived from C57Bl6 mice, three and ten days after CTX-induced muscle injury. DAPI nuclear counter staining is shown. Uninjured mice were used as negative control. Scale bar, 20 μm. (**f**) Quantification of the percentage of PJA1-positive cells per field in **e**. (**g**) Immunofluorescence analysis using antibodies against PJA1 on C2C12 cells cultured in GM or induced to differentiate for 24 h. DAPI counter staining is also shown. Scale bar, 50 μm. Data are represented as mean±s.e.m. of three independent biological replicates. *P<0.05; **P<0.01; ***P<0.001 (Student's test).

Ubiquitin is covalently attached to the substrate through a sequential reaction involving three different classes of enzymes (E1 activating enzymes, E2 ubiquitin conjugating enzymes and E3 ubiquitin ligases)^26^. To achieve substrate specificity the UPS relies on the last components of the chain, the E3 ubiquitin ligases, each of which targets only a small subset of proteins for degradation^27^. To identify the E3 ubiquitin ligase responsible for EZH2 degradation during muscle differentiation, we screened data sets from our previous transcriptome analysis performed in C2C12 cells^23^ for annotated and putative ubiquitin ligases^27^. Results shown in Supplementary Table 1 indicate that only a small number of E3 ubiquitin ligases are differentially expressed upon exposure to differentiation stimuli in C2C12 cells. We focused our attention on PJA1, as bioinformatics analysis of known and predicted protein-protein interactions using the String9.1 resource indicated that, of the up-regulated E3 ubiquitin ligases, only PJA1 was predicted to interact with EZH2, in agreement with previously published data^28^. qRT-PCR (Fig. 2a) and western blot analysis (Fig. 2b) confirmed PJA1 is induced during muscle differentiation. Furthermore, biochemical and functional analysis showed that *Pja1* is downstream of MYOD during the induction of the myogenic programme. First, chromatin immunoprecipitation (ChIP) analysis showed MYOD binds to a site upstream the *Pja1* promoter preferentially in C2C12 cells induced to differentiate (Fig. 2c). In addition, over-expression of *MyoD* in human fibroblasts is sufficient to up-regulate *Pja1* expression (Fig. 2d). Analysis of muscle regeneration *in vivo* showed that PJA1 is first observed in the cytoplasm of MYOD-positive cells three days after cardiotoxin (CTX)-induced muscle damage (Fig. 2e). At later stages of the regeneration process, PJA1 re-localizes to the nucleus of MYOD-positive cells and the number of PJA1-positive cells starts to decrease (Fig. 2e,f). This cytoplasm-to-nucleus switch also occurs when C2C12 muscle cells are induced to differentiate (Fig. 2g) and suggests that PJA1 may have different targets in proliferating myoblasts and committed myocytes. These results indicate that PJA1 is a novel component of the myogenic programme and point out to a temporal control of PJA1 nuclear activity during skeletal muscle regeneration.

### PJA1 induces EZH2 degradation in response to p38α activation

**Figure 3:**
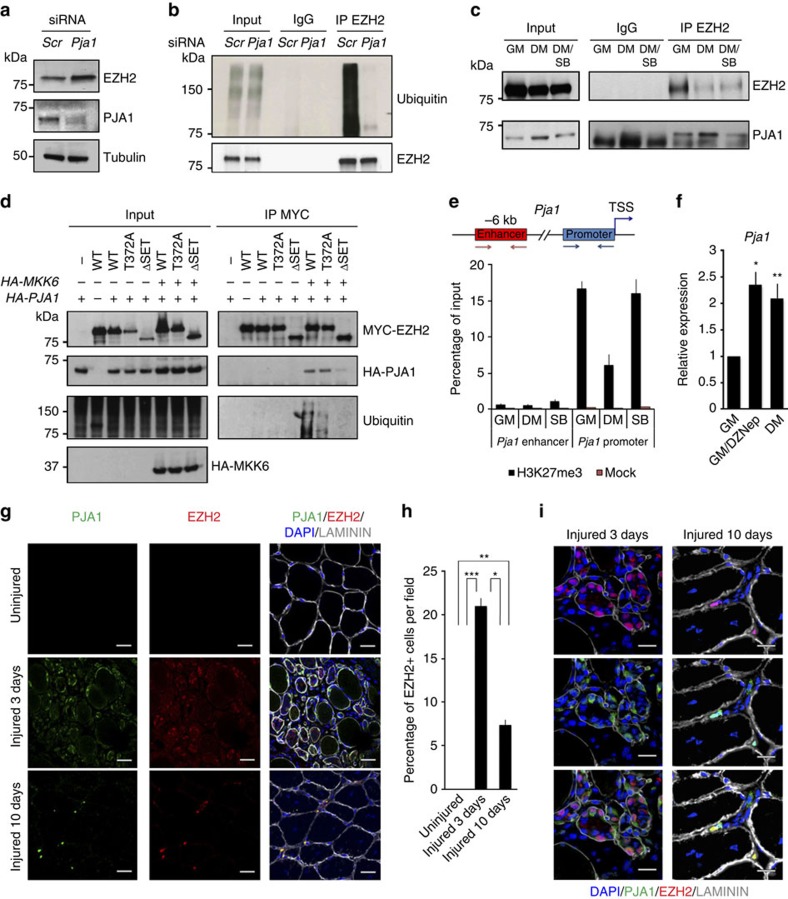
PJA1 marks EZH2 for proteasome-mediated degradation upon p38α activation. (**a**) Western blot showing EZH2, PJA1 and tubulin levels in C2C12 cells incubated in DM after transfection with control (*Scr*) or siRNA against *Pja1.* (**b**) Western blot showing ubiquitin associated to immunoprecipitated EZH2 and control IgG in C2C12 cells treated as in **a**. MG132 was added to the cells 2 h before collecting to avoid degradation of ubiquitinated EZH2. Input represents 2% of immunoprecipitated material. (**c**) Co-immunoprecipitation using an antibody against EZH2 performed in C2C12 cells cultured in GM or induced to differentiate in the absence (DM) or presence (DM/SB) of SB. IgG was used as a negative control. Western blot against EZH2 and PJA1 was used to visualize interacting proteins. Input represents 10% of immunoprecipitated material. (**d**) Co-immunoprecipitation using an anti-MYC antibody performed in HEK293 cells transfected with the HA-tagged *Pja1*, HA-tagged *MKK6EE* and the indicated myc-tagged *Ezh2* constructs. Figure shows western blot against endogenous ubiquitin and MYC and HA epitopes. Input represents 10% of immunoprecipitated material. (**e**) ChIP assay performed in C2C12 cells cultured in GM or induced to differentiate for 18 h in the absence (DM) or presence (SB) of SB 202190 using an antibody against H3K27me3. Mock IP is included as a negative control. Graph shows qPCR amplification of *Pja1* upstream regions on immunoprecipitated material normalized against input DNA. (**f**) qRT-PCR showing *Pja1* levels in C2C12 myoblasts cultured in the absence (GM) or presence (GM/dZNep) of dZNep as compared with cells induced to differentiate (DM). (**g**) Immunofluorescence using antibodies against EZH2, PJA1 and laminin on transversal sections of TA muscles derived from C57Bl6 mice, three and ten days after CTX-induced muscle injury. DAPI counter staining is also shown. Uninjured mice were used as negative control. Scale bar, 20 μm. (**h**) Quantification of the percentage of EZH2-positive cells per field in **g**. (**i**) Magnification of a representative image showing localization of PJA1 and EZH2 three and ten days after CTX-mediated muscle injury. Scale bar, 10 μm. Data are represented as mean±s.e.m. of three independent biological replicates. *P<0.05; **P<0.01; ***P<0.001 (Student's test).

We next investigated if PJA1 regulates EZH2 levels during muscle differentiation. siRNA-mediated depletion of the gene stabilizes EZH2 protein in C2C12 cells induced to differentiate (Fig. 3a) and impairs its ubiquitination as shown by immunoprecipitation of endogenous EZH2 followed by western blot against ubiquitin (Fig. 3b). However, the strong ubiquitination observed upon immunoprecipitation of EZH2 in cells transfected with control siRNA (Fig. 3b) suggests that EZH2-associated proteins may be also target of PJA1-induced ubiquitination in muscle cells. This is consistent with previous work from Ciechanover lab showing that the three main PRC2 components (EZH2, SUZ12 and EED) are target of this E3 ubiquitin ligase in a cell-free system^28^. Mass-spectrometry analysis in control and depleted cells would unequivocally address the role of PJA1 in regulating EZH2 ubiquitination in muscle cells and would allow to identify other putative targets.

To further dissect the functional interplay between PJA1, EZH2 and p38α in muscle cells we investigated if EZH2 and PJA1 directly interact upon induction of differentiation and the contribution of the p38α pathway to such interaction. Co-immunoprecipitation analysis, performed in the presence of the proteasome inhibitor MG132 to avoid degradation of PJA1-bound EZH2, demonstrated that the interaction between endogenous PJA1 and EZH2 increases when C2C12 cells are induced to differentiate and is partially blocked in the presence of SB202190 (Fig. 3c). Moreover, co-immunoprecipitation experiments in an heterologous system upon expression of myc-tagged *Ezh2* constructs alone or in combination with HA-tagged *MKK6EE* and HA-tagged *Pja1* demonstrated that activation of the p38α signalling is essential for EZH2/PJA1 interaction (Fig. 3d). Interestingly, they also showed that, despite being necessary for EZH2 ubiquitination, phosphorylation of T372 is dispensable for the interaction with PJA1 (Fig. 3d and Supplementary Fig. 1e,f), suggesting that additional phosphorylation events likely take place to regulate PJA1/EZH2 interaction in response to p38α activation. In addition, interaction with the ubiquitin ligase depends on the integrity of the SET domain (Fig. 3d), which explains the increased stability of the ΔSET mutant (Fig. 1e,f). Altogether, the data presented here indicate that activation of the p38α signalling is necessary for PJA1/EZH2 interaction and subsequent EZH2 degradation during muscle differentiation.

A detailed analysis of the expression profile *in vivo* showed that *Pja1* is first expressed three days after CTX-mediated muscle injury, whereas *Ezh2* expression starts at day two (Supplementary Fig. 2). This observation prompted us to investigate if EZH2 is involved in regulating *Pja1* expression before terminal differentiation. ChIP experiments showed enrichment of H3K27me3 at the promoter of *Pja1* in proliferating myoblasts (Fig. 3e). H3K27me3 levels decreased upon induction of differentiation and were partially recovered in the presence of SB202190. Moreover, treatment of proliferating C2C12 cells with the commonly used PRC2 inhibitor 3-Deazaneplanocin A (dZNep) anticipated *Pja1* expression (Fig. 3f). This suggests PRC2 represses *Pja1* expression in proliferating myoblasts, maybe as a safeguard mechanism to avoid premature EZH2 degradation. A second mechanism to avoid premature degradation of EZH2 is given by the different cellular localization of the proteins at early and late stages of the regeneration process. Immunofluorescence analysis on transversal muscle sections showed that, despite PJA1 staining is observed three days after CTX-induced muscle injury, the protein localizes in the cytoplasm of EZH2-positive cells. On the contrary, PJA1 is observed in the nucleus of EZH2-positive cells ten days after CTX injection, coincident with a decrease in the number of EZH2-positive cells (Fig. 3g–i). Furthermore, we detected some PJA1-positive, EZH2-negative cells at ten days, probably representing cells that have just down-regulated EZH2 (Fig. 3i). Altogether, data presented here suggest the existence of a self-limiting regulatory loop that controls PJA1 and EZH2 levels during muscle progenitors differentiation.

### PJA1 is essential for muscle differentiation

**Figure 4:**
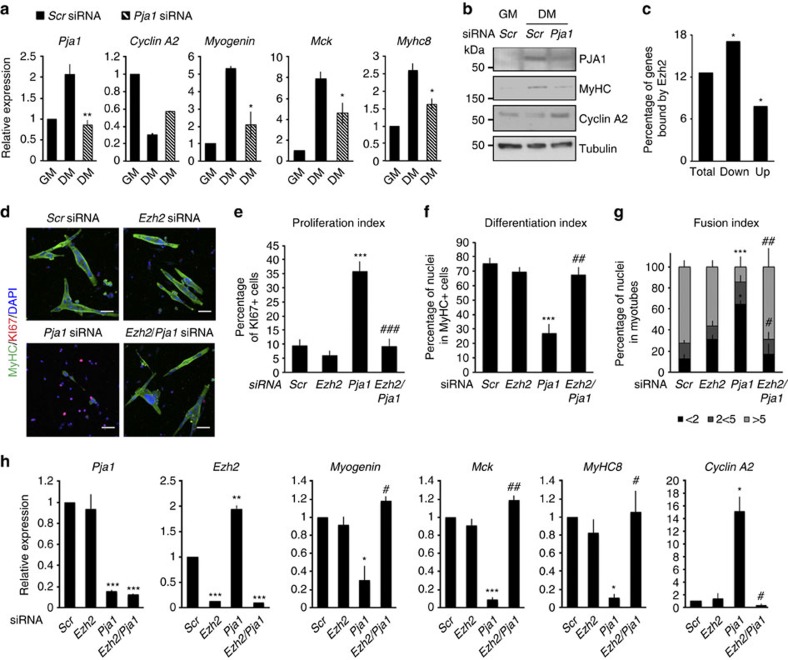
PJA1 induces terminal differentiation of muscle cells. (**a**) qRT-PCR showing the expression of muscle and cell-cycle related genes in C2C12 cells transfected with control (*Scr*) siRNA (black bars) or siRNA against *Pja1* (stripped bars) and incubated either in GM or DM. (**b**) Western blot showing PJA1, MyHC, cyclin A2 and tubulin levels in the same conditions as in **a**. (**c**) Graph showing the percentage of genes differentially expressed (both up- and down-modulated) upon siRNA-mediated depletion of *Pja1* that are bound by EZH2 in myoblasts^29^ (±20 kb from TSS). *<0.05 (hypergeometric test). (**d**) Immunofluorescence using antibodies against MyHC and KI67 on satellite cells transfected with control siRNA or siRNA against *Pja1* and *Ezh2*. DAPI counter staining is also shown. Scale bar, 40 μm. (**e**) Proliferation index calculated as the percentage of KI67-positive cells in **d**. (**f**) Differentiation index, calculated as the percentage of nuclei in MyHC-positive structures in **d**; (**g**) Fusion index, calculated as the percentage of nuclei in MyHC-positive structures containing more than five, between two and five or less than two nuclei per structure in **d**. (**h**) qRT-PCR showing the expression of muscle and cell-cycle related genes in cells treated as in **d**. The levels of *Ezh2* and *Pja1* in the different conditions are also shown as a control. Data are represented as mean±s.e.m. of three independent biological replicates. * in **e**–**h** indicate statistical significance versus *Scr* siRNA; # indicate statistical significance versus *Pja1* siRNA. */# *P*<0.05; **/## *P*<0.01; ***/### *P*<0.001 (Student's test).

To address the functional role of PJA1 during skeletal myogenesis we first performed siRNA-mediated depletion of the gene in C2C12 cells and we induced them to differentiate. The efficiency of knock-down was investigated by qRT-PCR (Fig. 4a) and western blot experiments (Fig. 4b). Biochemical analysis through qRT-PCR (Fig. 4a) and western blot (Fig. 4b) showed decreased expression of muscle markers and increased expression of genes necessary for cell-cycle progression. The strong block in the differentiation of muscle cells depleted of *Pja1* points to this MYOD-regulated E3 ubiquitin ligase as an essential component of the muscle differentiation programme. To confirm this hypothesis, we performed RNA-seq analysis on control and *Pja1*-depleted C2C12 cells. Ingenuity pathway analysis of the differentially expressed genes confirmed a decrease in the categories of ‘muscle formation' and ‘cell differentiation' as early as 24 h post-transfection of the siRNA (Supplementary Fig. 3a). We then intersected the expression data with available ChIP-seq data for EZH2 in muscle cells^29^. The hypergeometric test indicated that genes down-regulated in *Pja1*-depleted cells are significatively enriched in PRC2 targets (Fig. 4c), further suggesting PJA1 modulates muscle differentiation at least in part through EZH2 degradation.

We then investigated if PJA1 is also necessary for satellite cells differentiation and if it acts through regulation of EZH2 function. To this end we performed siRNA-mediated depletion of *Pja1*, alone or in combination with siRNA-mediated depletion of *Ezh2,* in satellite cells isolated from C57/Bl6 mice. Immunofluorescence analysis showed that depletion of *Pja1* impairs the formation of multinucleated myosin heavy chain (MyHC)-positive myotubes and increases the number of proliferating, KI67-positive, activated satellite cells as compared with control (scramble)-transfected cells. On the contrary, this differentiation defect is fully reverted in the double knock-down cells (Fig. 4d–g). qRT-PCR analysis confirmed the block of differentiation in the absence of *Pja1* and the reversed phenotype in the double knock-down cells (Fig. 4h). Interestingly, we noticed that *Ezh2* RNA levels are kept high in *Pja1*-depleted satellite cells (Fig. 4h), suggesting EZH2 protein degradation is an early event in the differentiation programme that precedes down-regulation of the gene by transcriptional and post-transcriptional mechanisms. Finally, to unequivocally address if increased EZH2 activity mediates the differentiation defect observed in *Pja1*-depleted cells, we treated cells transfected with either scramble siRNA or siRNA against *Pja1* with the EZH2 catalytic inhibitor GSK-126. Results shown in Supplementary Fig. 3b–d indicate that GSK-126 reverts the phenotypic defect observed in satellite cells depleted of *Pja1*. These results indicate that an accumulation of EZH2 protein and activity is the primary defect leading to impaired differentiation in the absence of *Pja1*.

### PJA1 pro-myogenic function depends on its catalytic activity

**Figure 5:**
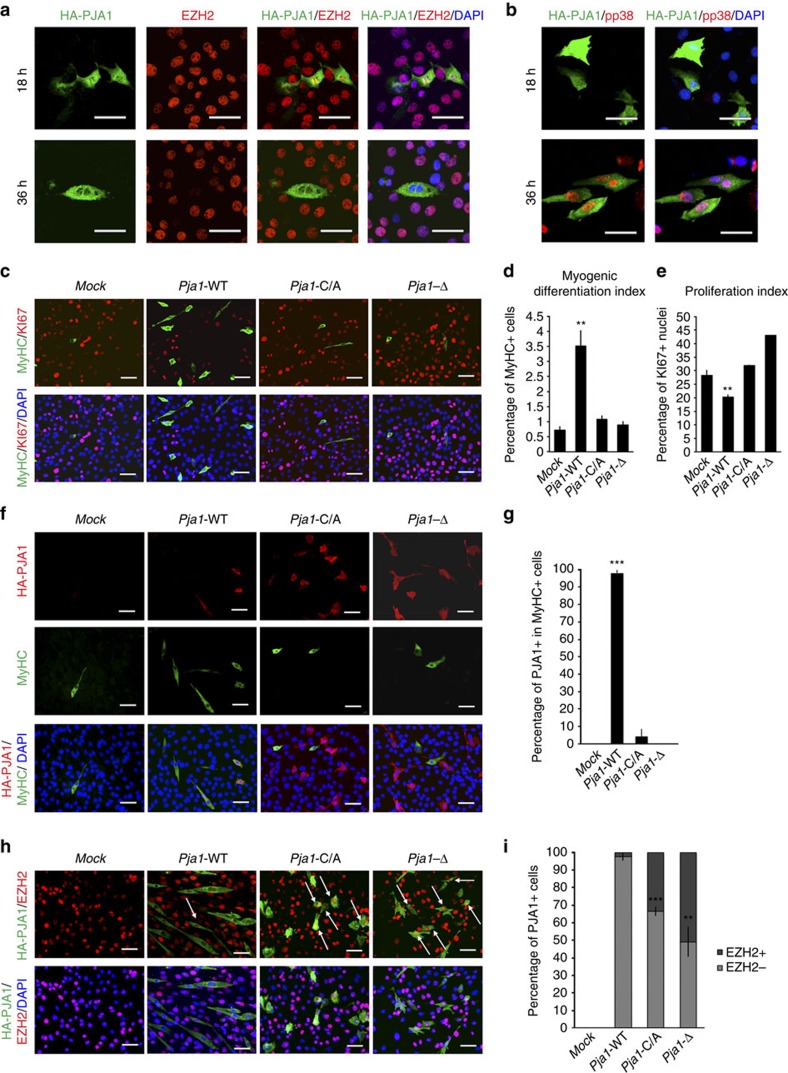
PJA1 catalytic activity is essential for its pro-myogenic function. (**a**) Immunofluorescence using antibodies against HA and EZH2 on C2C12 cells transfected with HA tagged wt *Pja1* and cultured in GM for 18 and 36 h. Scale bar, 50 μm. (**b**) Immunofluorescence using antibodies against HA and phospho-p38 (pp38) on C2C12 cells transfected with HA tagged wt *Pja1* and cultured in GM for 18 and 36 h. Scale bar, 50 μm. (**c**) Immunofluorescence using antibodies against MyHC and KI67 on C2C12 cells transfected with HA tagged wt *Pja1* or with mutants in the catalytic activity (C/A and Δ) and cultured in GM for 48 h. Scale bar, 40 μm. Quantification of the differentiation (**d**) and proliferation (**e**) index in **c**. (**f**) Immunofluorescence using antibodies against MyHC and HA on cells treated as in **c**. Scale bar, 40 μm. (**g**) Quantification of the percentage of HA-PJA1-positive cells in MyHC-positive structures. (**h**) Immunofluorescence using antibodies against HA and EZH2 on cells treated as in **c**. Scale bar, 40 μm. (**i**) Quantification of the percentage of HA-PJA1-positive cells per field contained within EZH2-positive or EZH2-negative cells. Data are represented as mean ±s.e.m. of four independent biological replicates. **P<0.01; ***P<0.001 (Student's test).

Finally, to investigate if the E3 ubiquitin ligase activity of PJA1 is necessary for EZH2 degradation and myogenic differentiation, we over-expressed HA-tagged *Pja1* constructs on C2C12 proliferating myoblasts and we measured the effects on differentiation and EZH2 levels by immunofluorescence experiments (Fig. 5a–g). We first confirmed the presence of over-expressed wt PJA1 in the nucleus of C2C12 cells 18 h after transfection. Interestingly, nuclear HA-PJA1 decreases after 36 h, coincident with p38α activation, EZH2 down-modulation and the appearance of multinucleated structures (Fig. 5a,b). Furthermore, our data show that over-expression of wt *Pja1* anticipates muscle differentiation in cells cultured in proliferation conditions (Fig. 5c–g). This effect is dependent on the catalytic activity of the protein, as constructs lacking the ubiquitin ligase domain (Δ) or containing a point mutation on the cytosine 353 (C353A)^30^ failed to induce the differentiation programme (Fig. 5c–g). Moreover, over-expression of wt *Pja1*, but not of the catalytically inactive mutants, correlates with a decrease in EZH2-positive nuclei (Fig. 5h,i). Altogether, our data indicate that signal-dependent degradation of EZH2 by PJA1 is a prerequisite for terminal differentiation of muscle cells.

## Discussion

EZH2 homeostasis is tightly regulated during cellular differentiation and alterations in EZH2 levels have been associated to tumour development in several tissues, including muscle^31^,^32^,^33^,^34^. Despite the enormous interest in this protein very little is known on its post-translational control^28^,^35^,^36^,^37^,^38^,^39^. EZH2 levels decrease during progenitor cells differentiation, being barely detectable in many adult specialized cells and tissues^5^. It was previously shown that several signalling pathways control EZH2 stability in response to damage or proliferation cues, including the Ataxia telangiectasia mutated kinase^35^ or cell-cycle dependent kinase 1 (ref. 36). However, the mechanism leading to protein degradation as cells enter the differentiation programme has remained largely unaddressed. Here we demonstrate that p38α kinase, which is activated by differentiation cues in muscle stem cells, marks EZH2 for proteasome-mediated degradation during skeletal myogenesis. Furthermore, we identified the Ring domain protein PJA1 as a differentiation-induced E3 ubiquitin ligase involved in the control of EZH2 levels. *Pja1* was first identified in mouse as a gene with homology to Drosophila melanogaster gene *goliath*, which is involved in the cell fate determination of mesodermal cells^40^. Work from Ciechanover lab recently showed that PJA1 targets the main PRC2 components, including EZH2, to proteasome-mediated degradation in cancer cells^28^. Consistently, treatment of tumour cells with the commonly used EZH2 inhibitor dZNep, which leads to degradation of PRC2 core components, selectively induces *Pja1* expression^41^. The data presented in this work suggest PJA1 is also the enzyme regulating EZH2 levels upon p38α activation in muscle cells. PJA1 is selectively induced upon induction of differentiation and siRNA-mediated depletion of the gene blocks myogenesis. Despite we do not present here direct mass-spectrometry evidence of PJA1-mediated ubiquitination of EZH2 and we cannot exclude PJA1 regulates other proteins during skeletal myogenesis, our data indicate that the pro-myogenic function of PJA1 involves EZH2 degradation, as shown by rescue experiments.

PJA1-mediated degradation of EZH2 is an early event in the myogenic programme and precedes down-regulation of the gene by transcriptional and/ or post-transcriptional mechanisms. Our data also suggest that several mechanism safeguard EZH2 from premature degradation in proliferating myoblasts, including the low levels of the E3 ubiquitin ligase PJA1, its predominantly cytoplasmic localization and the lower activity towards the unphosphorylated form of EZH2. Furthermore, analysis of available ChIP-seq data^29^ and our experiments indicate that EZH2 binds *Pja1* genomic region in proliferating myoblasts, suggesting that an auto-regulatory loop finely regulates the levels of EZH2 and its E3 ubiquitin ligase during myogenesis. In this scenario, activation of p38α by regeneration cues and subsequent phosphorylation of T372 of EZH2, may un-tip the balance in favour of EZH2 degradation, which in turn de-represses *Pja1* promoter that is then activated by the muscle regulatory factor MYOD. Despite our data do not exclude the possibility that p38β contributes to EZH2 phosphorylation, several lines of evidence point out to p38α as the main mediator of this pathway. First, our previous work showed that p38α phosphorylates EZH2 and this phosphorylation is necessary to repress *Pax7* expression and induce cell-cycle exit in satellite cells. Consistently, siRNA-mediated depletion of *p38α*, but not *p38β*, impaired Pax7 down-modulation^6^. Second, work from Munoz-Canoves lab using tissue-specific knock-out mice demonstrated that p38α, but not p38β-deficient myoblasts, show an impaired differentiation potential and continue to proliferate even in differentiation conditions^21^,^42^. Moreover, the same group recently showed *p38β* is barely expressed in differentiating myoblasts^24^. Altogether, published work and the evidence presented here strongly suggest that p38α is the kinase mediating EZH2 phosphorylation during skeletal muscle regeneration, leading to a switch of target genes and subsequent UPS-mediated degradation. The fact that the same signal regulates both activity and degradation has been previously described for some transcription factors and has been proposed as a mechanism to terminate transcriptional activation^43^,^44^. Here we propose that this regulatory mechanism can be extended also to chromatin modifiers, such as Polycomb proteins.

Interestingly, our results also point out to an unanticipated role of the E3 ubiquitin ligase PJA1 in the activation of a positive feedback loop that is initiated by the degradation of phosphorylated EZH2, as an early event associated to muscle differentiation that ultimately leads to the complete elimination of EZH2 in differentiated muscles. This is consistent with published data showing the existence of an auto-regulatory loop between EZH2 protein and *Ezh2*-targeting miRNAs, such as miR-214, in proliferating cells. Reduced amounts of EZH2 would alleviate miR-214 repression, which in turn targets *Ezh2* RNA^7^.

In summary, the data presented here point out to a key role for controlled proteolysis in the regulation of gene expression during terminal differentiation. These results add to recent reports on differentiation-associated down-regulation of basal transcriptional machinery components^45^ and other key proteins implicated in skeletal myogenesis^46^,^47^,^48^. Extensive work from the past decade has pointed out to a fundamental role of the UPS in regulating gene expression through different mechanisms, ranging from transcription initiation, elongation, RNA processing and chromatin modification (reviewed in refs 49, 50). Moreover, proteasome activity has been shown to regulate the function of RNA polymerase II and transcriptional activators^49^,^50^. Strong transcription factors often have a short half-life^51^,^52^, and targeting active transcription factors to proteasome-mediated degradation has been proposed as a mechanism to regulate transcription termination^53^. Recently the UPS has been also involved in regulating the stability of chromatin modifying enzymes^54^ and chromatin remodellers^55^, indicating that protein degradation contributes to set up the correct chromatin landscape for terminal differentiation. Here we unveil a novel mechanism through which the UPS remodels the chromatin during terminal differentiation via signal-dependent degradation of EZH2.

## Methods

### Mice and muscle injury

All the experiments were performed in 2–3 months old male C57/Bl6 mice. Mice were bred and maintained according to the standard animal facility procedures, and all experimental protocols were approved by the internal Animal Research Ethical Committee according to the Italian Ministry of Health and complied with the NIH Guide for the Care and Use of Laboratory Animals. For local muscle injury C57/Bl6 mice were anaesthetized via carbon dioxide inhalation and received 20 μl intramuscular injections of cardiotoxin (CTX, C9759 Sigma 10 μM) into the TA. Mice were killed 1, 2, 3, 5, 7, 10, 14 or 20 days post-injury, depending on the experiment.

### Satellite cell isolation and culture

Satellite cells were isolated by fluorescence-activated cell sorting. Hind limb muscles were minced and digested in Hank's balanced salt solution (HBSS) (Gibco) containing 2 mg ml^−1^ Collagenase A (Roche), 2.4 U ml^−1^ Dispase I (Roche), 10 ng ml^−1^ DNase I (Roche), 0.4 mM CaCl2 and 5 mM MgCl2 for 90 min at 37 °C. Cells were stained with primary antibodies for 30 min on ice. The following antibodies were used: CD31- efluor450 (eBioscience, cat. no. 48-0311, clone: 390, dilution 1:50), CD45-eFluor450 (eBioscience, cat. no. 48-0451, clone: 30-F11, dilution 1:50), Ter119-eFluor450 (eBioscience, cat. no. 48-5921, clone: TER-119, dilution 1:50), Ly-6A/E (Sca1)-FITC (eBioscience, cat. no. 11-5981, clone: D7, dilution 1:50) and α7integrin-647 (AbLab, cat. no. 67-0010-10, clone R2F2, dilution 1:500). Cells were finally washed and suspended in HBSS containing 0.2% w/v BSA and 1% v/v Penicillin–Streptomycin. Flow cytometry analysis and cell sorting were performed on a DAKO-Cytomation MoFlo High Speed Sorter. Cells were plated on gelatin (0,1%, Stem cell Technologies) -coated dishes with GM (20% FBS, 10% horse serum, 1% chick embryo extract in DMEM). After 4–5 days, the medium was replaced with DM (DMEM supplemented with 2% HS and 0.5% chick embryo extract).

### Cell lines and treatments

C2C12 myoblasts, HEK293T cells and BJ fibroblasts (obtained from the ATCC and tested for mycoplasma contamination) were propagated in GM (DMEM supplemented with 10% FBS). After 2-3 days cells were induced to differentiate in DM (DMEM supplemented with 2% HS). Where indicated, cells were treated with SB202190 (Calbiochem, 5 μM), MG132 (Sigma, 10 μM), cycloheximide (CHX, Sigma, 20 mg ml^−1^), GSK-126 (BioVision, 4 μM) and dZNep (BioVision, 5 μM).

### Transfection and adenoviral infection

Transient transfections were performed using lipofectamine 2000 (Invitrogen) following manufacturer conditions. *Ezh2WT*, *Ezh2T372A* and *Ezh2ΔSET* myc-tagged pcDNA3 plasmids were described in ref. 6, whereas HA-pcDNA3-*MKK6EE* was described in ref. 56. Myc-tagged *Ezh2T372D* was obtained by site directed mutagenesis employing the Quickchange mutagenesis kit (Stratagene) following the manufacturer's instructions. Primers sequences are indicated in Supplementary Table 2. HA-*Pja1*, HA-*Pja1Δ* and HA-*Pja1C353A* pcDNA3 plasmids were kindly provided by Dr. Watanabe and have been previously described in ref. 30. HA-tagged pcDNA3-*Ubiquitin* was kindly obtained from Dr. Barilà and is described in ref. 57.

For adenoviral infection the viruses were amplified by transfection of HEK293 packaging cells and C2C12 and BJ cells were infected with control, *AdMKK6EE* or *AdMyoD* for 1 hr in serum-free medium before being placed in GM or DM.

### RNA interference

Down-regulation of *Pja1* and *Ezh2* expression in C2C12 and satellite cells was achieved by siRNA using ON-TARGETplus SMARTpool - mouse PJA1, SMARTpool- mouse EZH2 or ON-TARGETplus Non-targeting Pool (Dharmacon) as a control. RNA interference was performed according to Dharmafect3 (Dharmacon) transfection protocol.

### Western blot

Western blot on total cells was performed after lysis in 50 mM Tris-HCl (pH 7.6), 150 mM NaCl, 5 mM EDTA, 1% Triton X-100 supplemented with 1 mM PMSF and protease inhibitor mix. The following antibodies were used: MyHC (MF20, DSHB; dilution 1:100), cyclin A2 (Santa Cruz, SC-596; dilution 1:500), custom-made phospho-EZH2 (Primm Biotech, dilution 1:100), EZH2 (AC22, Cell Signaling; dilution 1:1,000), PJA1 (Proteintech, 17687-1-AP, dilution 1:1,000), Polyubiquitin (D9D5, Cell Signalling; dilution 1:1,000), Tubulin (Cell Signalling, 3873; dilution 1:1,000), MYC (Santa Cruz, SC-789 and SC-40; dilution 1:500) and HA (Santa Cruz, SC-805 and SC-7392; dilution 1:500). Uncropped scans of western blots presented in main figures are provided in Supplementary Fig. 4.

### Immunofluorescence

Frozen TA muscles were cut transversally, fixed in 4% PFA for 20 min and permeabilized with methanol for 6 min at −20 °C. Muscle sections were then blocked with a solution containing 4% BSA in PBS followed by incubation with anti-mouse AffiniPure Fab fragment (Jackson) to avoid unspecific binding. Immunostaining with primary antibodies was performed overnight at 4 °C. Secondary antibodies coupled to Alexa Fluor 488 or 594 (Molecular Probes) were used to reveal antibody binding. Nuclei were visualized by counter staining with DAPI. Cultured cells were fixed in 4% PFA, permeabilized with either 0.25% Triton (for phopho-p38 antibody) or methanol and blocked with 4% BSA in PBS before antibody incubation. Immunostaining and detection was performed as above. Primary antibodies used were: anti-laminin (Sigma, L9393; dilution 1:1,000), anti-PJA1 (Proteintech, 17687-1-AP; dilution 1:50), anti-MYOD (BD, 554130; dilution 1:50), anti-EZH2 (AC22, Cell Signaling; dilution 1:150), anti-MyHC (MF-20, DSHB; dilution 1:30 and Santa Cruz, SC-20641; dilution 1:100), anti-KI67 (BD 556003; dilution 1:1.000), anti-HA (Santa Cruz, SC-805 and SC-7392; dilution 1:50) and anti-phospho-p38 (Cell Signalling, D3F9; dilution 1:150). Images were acquired with a Leica confocal microscope and edited using the Photoshop CS4 software. Fields reported are representative of all examined fields.

### Co-immunoprecipitation and ubiquitination analysis

Endogenous and exogenous Co-IPs were performed on total cell extracts by standard, non-denaturing, procedures. In brief, cells, pre-treated with MG132 for 2 h prior collecting, were lysed in 50 mM Tris-HCl (pH 7.5), 150 mM NaCl, 1% NP-40 supplemented with 1 mM PMSF and protease inhibitor mix. Cell extracts were precleared with protein G agarose for 1 hr at 4 °C, immunoprecipitated with 2 μg of anti-EZH2 (Diagenode, pAb-039-050), 3 μg of anti-MYC (Santa Cruz, SC-40) antibodies or 2–3 μg of normal rabbit IgG (Santa Cruz, SC-2027) for 3 h at 4 °C, and incubated with protein G agarose. Immunoprecipitates were extensively washed with the lysis buffer, resuspended in Laemmli buffer, separated on polyacrylamide gels and transferred to nitrocellulose membranes. Precipitated proteins were revealed by western blot with EZH2 (AC22, Cell Signaling), PJA1 (Proteintech, 17687-1-AP), Polyubiquitin (D9D5, Cell Signalling), MYC (Santa Cruz, SC-40) and HA (Santa Cruz, SC-805) antibodies.

### qRT-PCR

RNA was extracted with Trizol and retro-transcribed using the cDNA reverse transcription kit (Applied Biosystems) following manufacturer indications. Quantitative PCR was performed using the SYBR Green Master mix (Applied Biosystems). Gene expression was normalized to expression of the housekeeping gene GAPDH. Primers sequences are indicated in Supplementary Table 2.

### Chromatin immunoprecipitation

Chromatin was cross-linked for 12 min with 1% formaldehyde (Sigma) and glycine was then added to a final concentration of 0.125 M for 5 min. After washing and collecting the cells, samples were incubated in nuclei lysis buffer (50 mM Tris-HCl pH 8.1, 1% SDS, 10 mM EDTA) on ice for 30 min. Sonication to obtain chromatin fragments of around 200–300 bp was performed using a Bioruptor UCD-200 sonicator (Diagenode). Chromatin extracts were immunoprecipitated overnight on a rotating platform at 4 °C with 3 μg the following antibodies: anti-MyoD (Santa Cruz, sc-760) and anti-trimethyl-histone H3 (Lys27) (Millipore 07-449). Normal rabbit IgG was used as negative control (mock). Immunoprecipitated chromatin was conjugated with G-protein magnetic Beads (Invitrogen). After extensive washing, bound DNA fragments were eluted and analysed by quantitative PCR using the SYBR Green Master Mix (Applied Biosystems). Primers sequences are indicated in Supplementary Table 2.

### RNA-seq

RNA was collected using Trizol reagent and RNA libraries were prepared for sequencing using standard Illumina protocols (4-plex, HiSeq2000 1 × 50 bp). RNA-Seq reads were mapped using TopHat-2.0.8br (ref. 58) allowing up to four mismatches per read. Read count in mus musculus GRCm38.78 genes was performed with HTSeq-0.6.1p1 (ref. 59). Differential expression analysis was performed with DESeq^60^. Genes were considered differentially expressed if the *P* value was<0.05 and the fold change was lower than 0.83 or higher than 1.2. Ingenuity pathway analysis (QIAGEN Redwood City, www.qiagen.com/ingenuity) was used for gene ontology.

### ChIP-seq analysis

File with mapped EZH2 ChIP-Seq reads was downloaded from GSM628019. Input file was kindly provided by Dr Sartorelli. Peaks were called using MACS2 2.1.0 (ref. 61) with *P* value 10-3. EZH2 peak coordinates were converted from 9 mm to 10 mm using CrossMap-0.1.6 (ref. 62). ChIPpeakAnno^63^ was used to annotate GRCm38.p3 ENSEMBL genes to EZH2 peaks. Comparisons with RNA-seq data were made using the hypergeometric test.

### Statistical analysis

Power analysis based on previous studies and assuming a normal distribution of the samples, s.d. equal to 20–25%, *δ* equal to 50–60%, type 1 error (*α*) equal to 5% and power (1-*β*) equal to 80% indicated a sample size of three control and three experimental subjects. Data are presented as mean±s.e.m. of at least three independent biological replicates as indicated in each figure. Comparisons were made using the Student's *t*-test assuming a paired two-tailed distribution, with significance being defined as *P*<0.05 (*/#), *P*<0.01 (**/##) and *P*<0.001 (***/###).

### Data availability

RNA-Seq raw data have been uploaded in NCBI's Sequence Read Archive (SRA https://submit.ncbi.nlm.nih.gov/subs/sra/ ), BioProject ID: PRJNA344797 (https://www.ncbi.nlm.nih.gov/bioproject/344797), BioSample accession numbers: SAMN05838210 (https://www.ncbi.nlm.nih.gov/biosample/5838210), SAMN05838211 (https://www.ncbi.nlm.nih.gov/biosample/5838211), SAMN05838212 (https://www.ncbi.nlm.nih.gov/biosample/5838212), SAMN05838213 (https://www.ncbi.nlm.nih.gov/biosample/5838213).

## Additional information

**How to cite this article:** Consalvi, S. *et al*. Praja1 E3 ubiquitin ligase promotes skeletal myogenesis through degradation of EZH2 upon p38α activation. *Nat. Commun.* **8,** 13956 doi: 10.1038/ncomms13956 (2017).

**Publisher's note:** Springer Nature remains neutral with regard to jurisdictional claims in published maps and institutional affiliations.

## Supplementary Material

Supplementary Information Supplementary Figures 1-4 and Supplementary Tables 1-2.
