# Supplementary material for: Praja1 E3 ubiquitin ligase promotes skeletal myogenesis through degradation of EZH2 upon p38α activation
Source: Nat Commun. 2017 Jan 9;8:13956. doi: 10.1038/ncomms13956 (PMC5423270; doi:10.1038/ncomms13956)
Supplement: Supplementary Information — Supplementary Figures 1-4 and Supplementary Tables 1-2. [file ncomms13956-s1.pdf]

Supplementary Figures

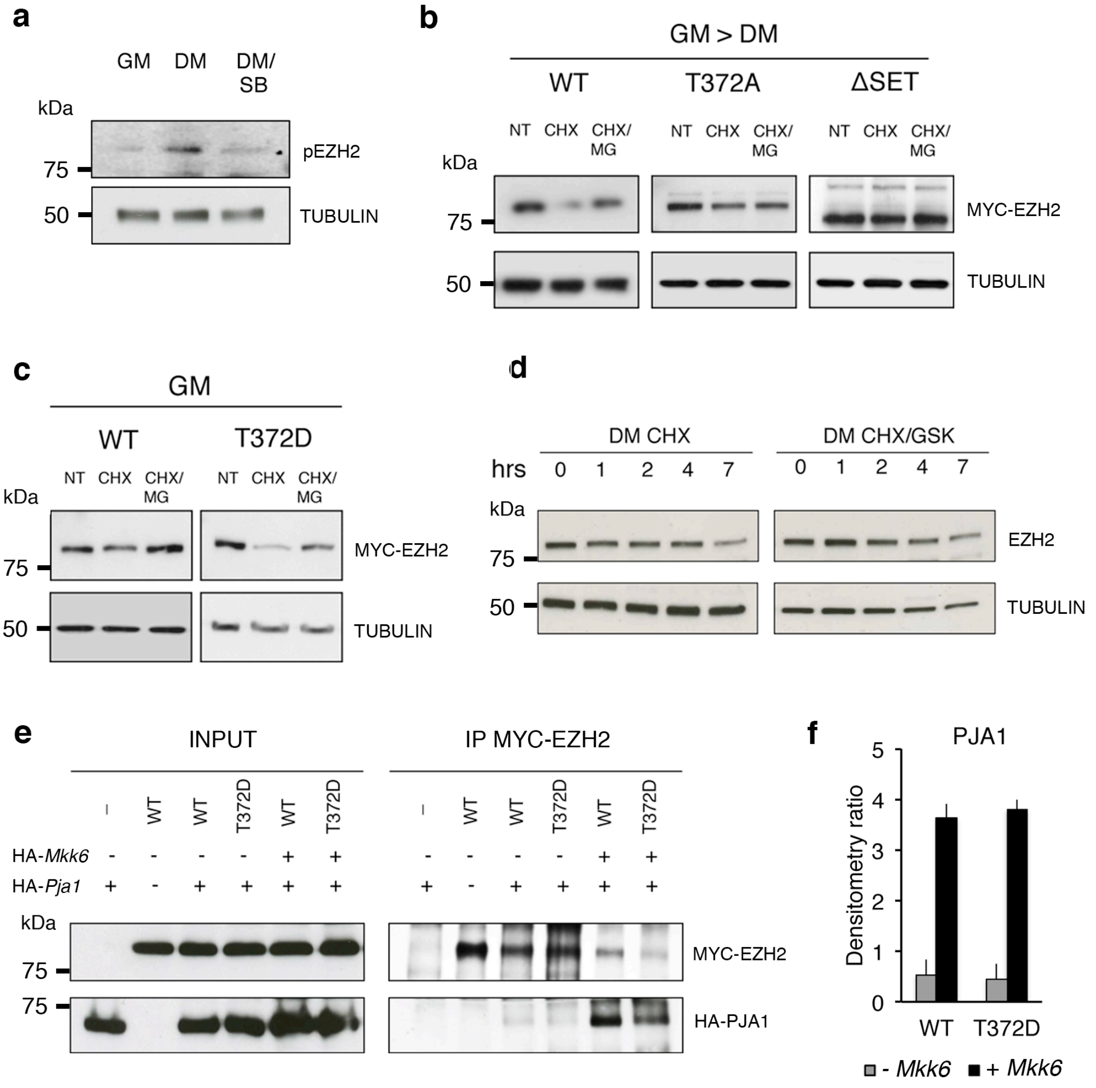

Supplementary Fig. 1.- EZH2 degradation requires p38α-mediated phosphorylation and the integrity of the SET domain. a) Western blot using a phospho-EZH2 antibody (phospho-T372) in C2C12 cells cultured in GM or after 12 h incubation in DM. TUBULIN is shown as a loading control. **b)** Western blot analysis of EZH2 levels in C2C12 cells transfected with either wt, phospho-mutant (T372A) or catalytically inactive (ΔSET) versions of myc-tagged *Ezh2* constructs and induced to differentiate (GM>DM) in absence (NT) or presence of cycloheximide (CHX) and the proteasome inhibitor MG132 (MG). TUBULIN is shown as a loading control. **c)** Western blot analysis of EZH2 levels in C2C12 cells transfected with either wt or phospho-mimic (T372D) versions of myc-tagged *Ezh2* constructs in proliferating

medium (GM) in absence (NT) or presence of cycloheximide (CHX) and the proteasome inhibitor MG132 (MG). TUBULIN is shown as a loading control. **d)** Western blot analysis of EZH2 levels in C2C12 cells induced to differentiate for the indicated hours with cycloheximide (DM CHX) and the EZH2 inhibitor GSK-126 (DM CHX/GSK). TUBULIN is shown as a loading control. **e)** Co-immunoprecipitation using an anti-MYC antibody performed in HEK293 cells transfected with the HA-tagged *Pja1*, HA-tagged *Mkk6EE* and the indicated myc-tagged *Ezh2* constructs. MG132 was added to the cells 2 hours before harvesting. Figure shows western blot against MYC and HA epitopes. **f)** Graph shows quantification of the relative PJA1 binding to the MYC-EZH2 constructs in e. Data are represented as mean +/- s.e.m. of two independent biological replicates.

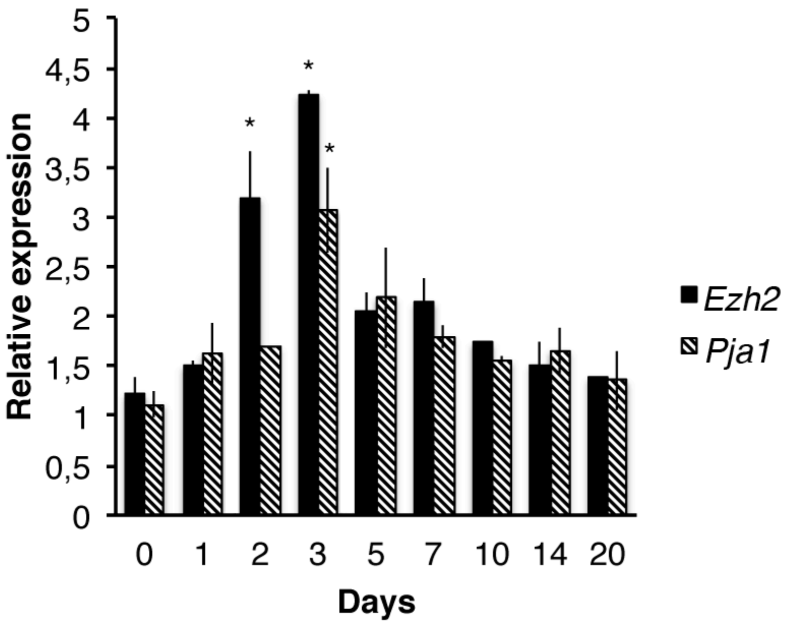

Supplementary Fig. 2.- *Pja1* and *Ezh2* expression profile during muscle regeneration. qRT-PCR data showing *Pja1* and *Ezh2* expression in regenerating muscle at the indicated days post CTX-mediated muscle injury. Data are represented as mean +/- s.e.m. of three independent biological replicates. *$p<0.05$ (Student's test).

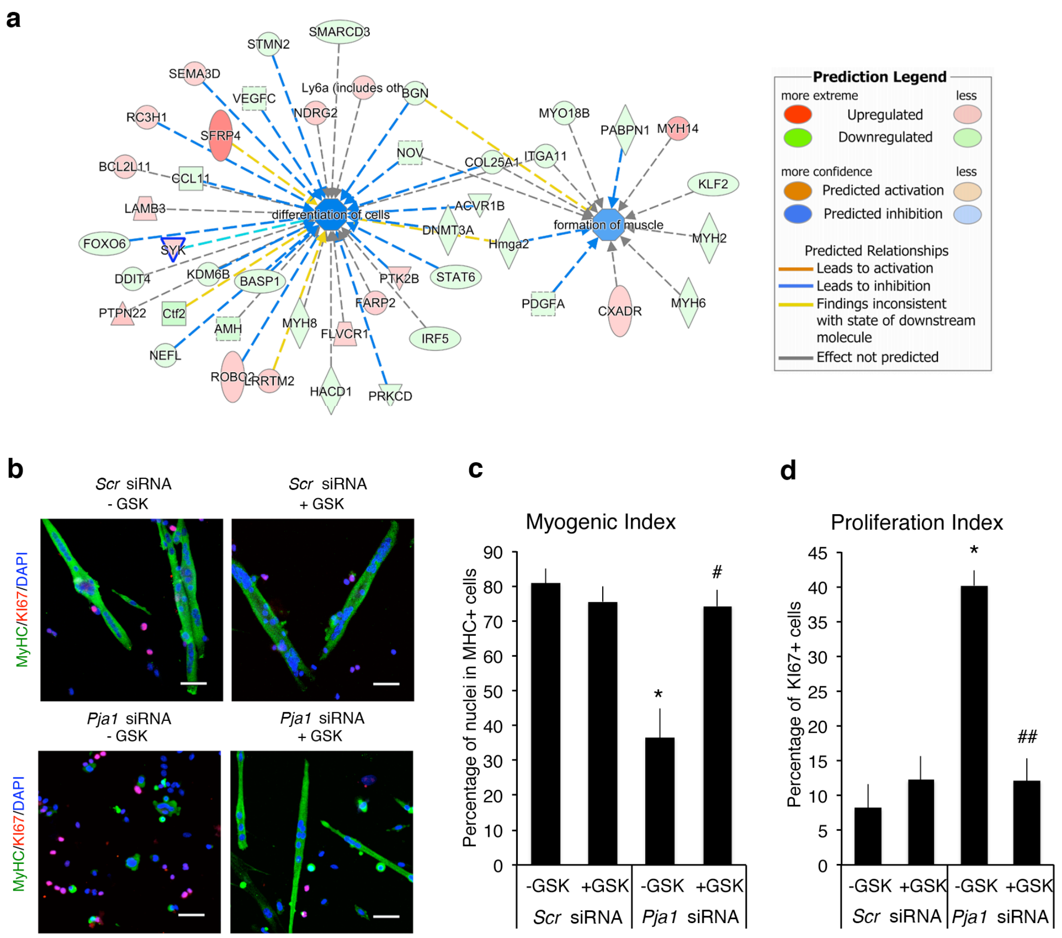

Supplementary Fig. 3.- *Pja1* depletion impairs muscle differentiation through a mechanisms involving EZH2 catalytic activity. a) IPA analysis of the differentially expressed genes upon siRNA-mediated depletion of *Pja1* in C2C12 showing predicted inhibition of the categories of "differentiation of cells" and "formation of muscle". **b)** Immunofluorescence using antibodies against MyHC and Ki67 on satellite cells transfected with control siRNA or siRNA against Pja1 and treated with the EZH2 catalytic inhibitor GSK-126. DAPI counter-staining is also shown. Scale bar = 40 μm **c)** Graph showing the differentiation index, calculated as the percentage of nuclei in MyHC-positive structures in b. **d)** Proliferation index calculated as the percentage of KI67-positive cells in b. Data are represented as mean +/- s.e.m. of three independent biological replicates. * indicate statistical significance versus *Scr* siRNA; # indicate statistical significance versus *Pja1* siRNA. */# $p<0.05$; **/## $p<0.01$ (student's test).

Fig. 1a

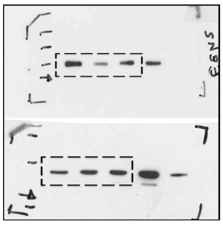

Fig. 1c

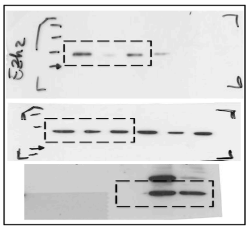

Fig. 1e

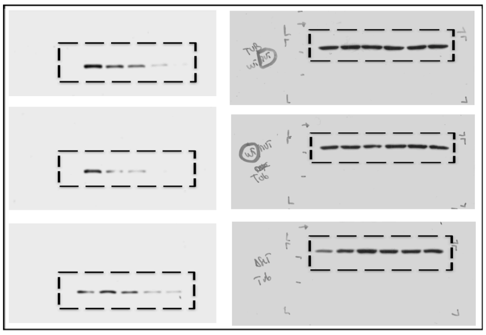

Fig. 1g

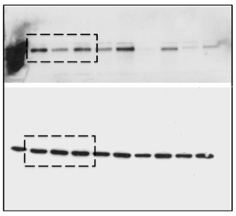

Fig. 1h

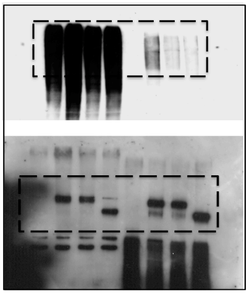

Fig. 2b

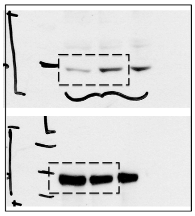

Fig. 3a

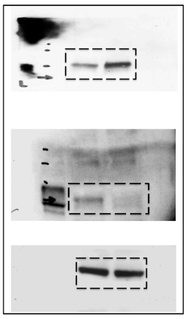

Fig. 3b

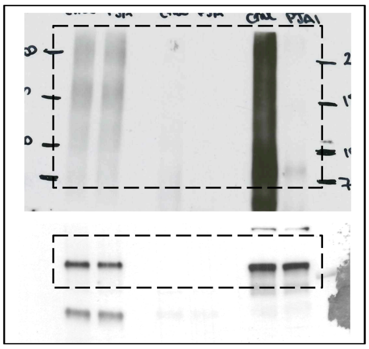

Fig. 3c

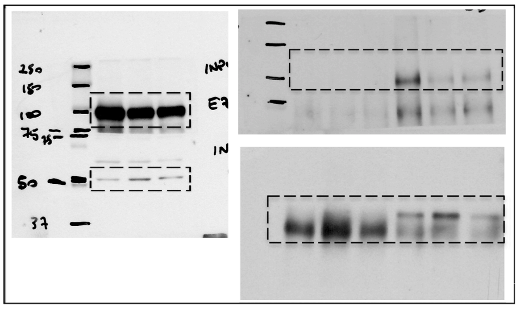

Fig. 3d

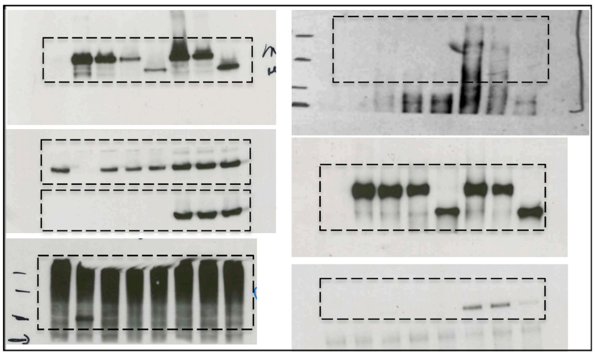

Fig. 4b

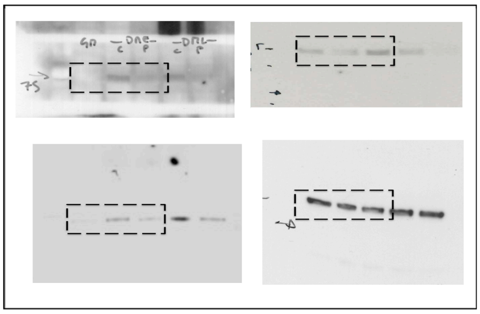

Supplementary Fig. 4.- Uncropped images of western blot figures shown in the main paper.

Supplementary Tables

E3-Ub-ligases modulated upon C2C12 differentiation			
UP-REGULATED		DOWN-REGULATED	
E3-Ub-ligase	FOLD	E3-Ub-ligase	FOLD
Asb2	14,4	Rfwd3	-1,6
Neurl	6,6	Hace1	-1,6
Trim72	4,8	Mid2	-1,5
Ttc3	3,4	Fbxl14	-1,5
Traf4	2,5		
Dtx4	2,2		
Fbxl6	2,1		
Pja1	2,0		
Rnf135	1,8		
Map3k1	1,7		
Birc2	1,7		
Pja2	1,7		
Rnf167	1,6		
Fbxop2	1,5		
zbtb24	1,5		

Supplementary Table 1.- E3 ubiquitin ligases in muscle cells. Table summarizing the E3 ubiquitin ligases modulated upon incubation of C2C12 cells in DM for 18 hours. Data were obtained by matching our previous transcriptome analysis in muscle cells with a list of known and putative E3 ubiquitin ligases.

Mutagenesis	
Ezh2 T372D	*Fwd:* AGTAGCAGGCCCAGC**GAC**CCCACCATTAATGTGC<br>*Rev:* GCACATTAATGGTGGG**GTC**GCTGGGCCTGCTACT
**RT-PCR**	
Ezh2	*Fwd:* CTAATTGGTACTTACTACGATAACTTT<br>*Rev:* ACTCTAAACTCATACACCTG TCTACAT
Pja1	*Fwd:* CATTTGCCAGCACACGTC<br>*Rev*: GGAATAATCCTCACTCTCAACAGC
CyclinA2	*Fwd*: CCCTGCA TTTGGCTGTGAAC<br>*Rev:* GGTGCTCCA TTCTCAGAACC
Myog	Fwd: GGCTCAAGAAAGTGAATGAGGC<br>Rev: CGATGGACGTAAGGGAGTGC
Mck	*Fwd:* AGTCCTACACGGTCTTCAAGG<br>*Rev:* AGGAAGTGGTCATCAATGAGC
MyHC8	*Fwd:* GAACTTGAAGGAGAGGTCGA<br>*Rev:* GAGCACATTCTTGCGGTCTT
Gapdh	*Fw:* CACCATCTTCCAGGAGCGAG<br>*Rev:* CCTTCTCCATGGTGGTGAAGAC
**ChIP**	
Pja1 Enhancer	*Fwd:* GGCTTGGGGTTCTATGGCTT<br>*Rev:* AAAGCAACGGTGAATGGCA
Pja1 Promoter	*Fwd:* GTGCTGCCTGTAATGAACG<br>*Rev:* AGTGAAATCTGGTGCTGTT

Supplementary Table 2.- Primers sequences. Table containing the sequences of primers used in this study.
